# Case report: PD-L1-targeted high-affinity natural killer cells and IL-15 superagonist N-803-based therapy extend overall survival of advanced metastatic pancreatic cancer patients

**DOI:** 10.3389/fonc.2025.1472714

**Published:** 2025-01-29

**Authors:** Tara Seery, Lennie Sender, Omid Jafari, Frank Jones, Patricia Spilman, Sandeep B. Reddy, Patrick Soon-Shiong

**Affiliations:** ^1^ Chan Soon-Shiong Institute for Medicine, El Segundo, CA, United States; ^2^ ImmunityBio Inc., Culver City, CA, United States; ^3^ Medical Imaging Center of Southern California, Santa Monica, CA, United States; ^4^ NantCell, Culver City, CA, United States

**Keywords:** advanced metastatic pancreatic cancer, 3rd line therapy, orchestrated, multi-modal, N-803, PD-L1 t-haNK cells, low dose chemotherapy

## Abstract

**Background:**

Metastatic pancreatic cancer (mPC) is an aggressive form of cancer with a poor prognosis and few therapeutic options after failure of the second-line treatment. Induction of immunogenic cell death (ICD) by use of relatively low-dose chemo- or radiation therapy, enhancement of immune responses by the IL-15 superagonist N-803 (Anktiva^®^), and targeting of programmed death receptor ligand 1 (PD-L1)-expressing cells may offer a therapeutic approach to refractory mPC with the potential to increase overall survival (OS).

**Methods:**

From late 2019 to 2021, single-patient Investigational New Drug (spIND) protocols for five mPC patients were designed and approved that generally comprised combined Abraxane (nab-paclitaxel) and gemcitabine therapy with experimental therapeutics N-803, PD-L1-targeted high-affinity natural killer (PD-L1 t-haNK) cells, and aldoxorubicin, a serum albumin-binding doxorubicin prodrug. Some patients also received stereotactic body radiation therapy (SBRT), cyclophosphamide, pembrolizumab, nivolumab, and/or experimental ETBX-051 (brachyury) and/or ETBX-061 (MUC1) vaccines. Duration of spIND treatment and responses, for some patients including imaging and carbohydrate antigen 19-9 (CA19-9) levels, and OS from initial diagnosis and the start of spIND therapy were assessed.

**Findings:**

The line/duration of spIND therapy was, for patients 1 through 5, respectively, second line/6.4 months, sixth line/3.5 months, third line/25.4 months, third line/7.4 months, and fourth line/23.2 months. OS from the commencement of spIND therapy was 13, 4.8, 26.9, 9, and 23.2 months, and OS from diagnosis was 22, 21, 42, 13, and 33 months for patients 1 through 5, respectively.

**Conclusions:**

The OS from the initiation of spIND for all patients exceeded the reported OS for the greater-than-second-line mPC patients and, for four of five patients, second-line therapy. The OS of 13, 26.9, and 23.2 months for three patients is particularly notable. The findings here support the ongoing clinical investigations of N-803 and PD-L1 t-haNK cells in combination therapy.

## Introduction

Pancreatic cancer has one of the highest rates of mortality worldwide (1) and is anticipated to become the second most common cause of death related to cancer by 2030 (2). At diagnosis, the 5-year survival rate of patients with metastatic pancreatic cancer (mPC) is only 14.4% for locally advanced disease and only 3% for those with distant metastases (3, 4); this rate decreases further for patients for whom first- and second-line therapies fail. Little survival data are available for third-line therapy, but as reported by Nagrial et al. (5) in their systematic review of locally advanced or mPC patient response to second-line treatment, the median overall survival (OS) was 4.0–5.4 months.

The current standard of care (SoC) for mPC includes FOLFIRINOX or Nab-paclitaxel plus gemcitabine as first-line therapy and crossover to Nab-paclitaxel/gemcitabine or FOLFIRINOX as second-line therapy (6–8). Nanoliposomal irinotecan with 5-fluorouracil (5-FU) and leucovorin is also approved as second-line therapy after gemcitabine therapy, based on the findings from the phase 3 NAPOLI 1 trial wherein the median survival was 6.1 months (9, 10). These treatment regimens are typically alternated for patients whose status allows for continued therapy and who do not opt for palliative care only.

There are little published data on OS after third-line therapy. In a study performed in Japan, an OS of 4.6 months for erlotinib plus gemcitabine as the third-line or greater therapy for mPC was reported (11). In a retrospective analysis of the efficacy of second-line or greater therapy for mPC patients, Bachet et al. reported that median OS was 4.6 months after first-line therapy only and 11.5 months from diagnosis and 4.7 months from initiation of second-line therapy for patients receiving two or three lines of therapy (12). These data highlight the unmet need for new therapeutic options for mPC patients beyond second-line therapy.

**Figure 1:**
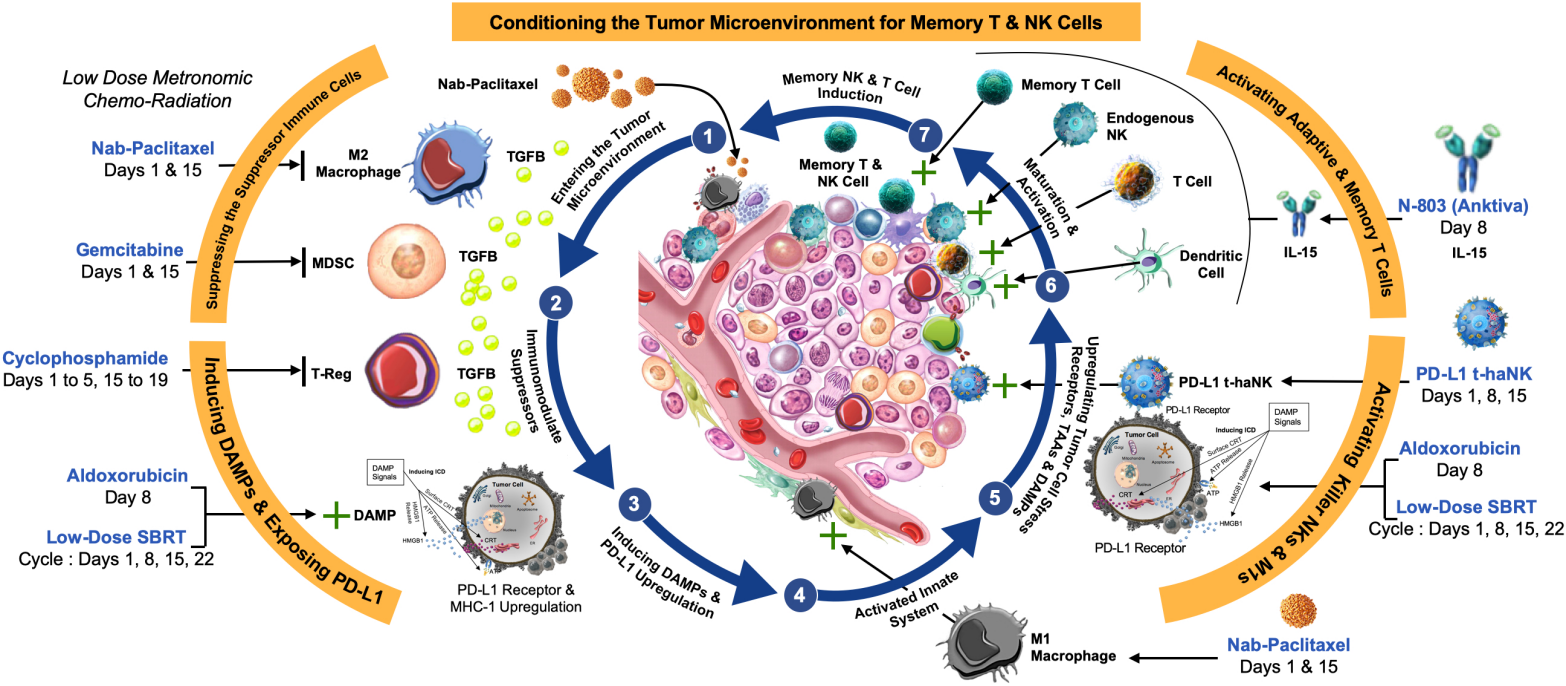
Rationale for multimodal therapy to establish immune memory. (1) Low-dose chemotherapy such as Nab-paclitaxel (Abraxane) and stereotactic body radiation therapy (SBRT) is anticipated to (2) reverse immunosuppression by M2 macrophages, myeloid-derived suppressor cells (MDSCs), and regulatory T cells (Tregs) in the tumor microenvironment (TME). (3) The release of damage-associated molecular patterns (DAMPs) from dying tumor cells leads to upregulation of MHC-1, and often PD-1/PD-L1 expression, (4) activating the innate immune system. (5) Further upregulation of cell stress receptors and tumor antigens by aldoxorubicin and SBRT also contributes to activation of M1 macrophages, cytotoxic T, and natural killer (NK) cells, an effect that is enhanced by (6) the IL-15 superagonist N-803. PD-L1-targeted high-affinity NK (PD-L1 t-haNK) cells target and reverse immunosuppression. The goal is (7) dendritic cell (DC) maturation and activation, leading to establishment of memory T cells.

Here, as an alternative therapeutic approach targeted to induce the death of cancer cells, we sought to enhance immunogenic cell death (ICD). In the tumor microenvironment (TME), ongoing expression of damage‐associated molecular patterns (DAMPs) should, in the presence of an effective immune response, elicit the release of ligands and expression of dendritic cell (DC) receptors that facilitate antigen processing and presentation, ultimately stimulating the anti-tumor cell cytotoxicity of T cells (13) and natural killer (NK) cells (14), with the goal of establishing immune memory (**Figure 1**).

Frequently, in the case of an ineffective immune response to neoplastic growth, expression of receptors such as the programmed death receptor 1 (PD-1) or its ligand (PD-L1) by immune cells in the TME circumvent an effective immune response and allow tumor cells to proliferate unabated. To re-initiate immune activity, inhibitors of PD-1 or PD-L1 (checkpoint inhibitors) are used.

In the therapeutic protocols described here, we used ImmunityBio’s investigational PD-L1-targeted high-affinity NK cells (PD-L1 t-haNK), a novel NK cell line that are NK-92 cells engineered to express high-affinity CD16, endoplasmic reticulum-retained interleukin (IL)-2, and a PD-L1-specific chimeric antigen receptor (CAR) (15, 16).

To further enhance the immune response, ImmunityBio’s interleukin-15 (IL-15) superagonist N-803 (nogapendekin alfa inbakicept, Anktiva^®^; previously known as ALT-803) (17), a human IL-15 variant bound to a dimeric human IL-15 receptor α (IL-15Rα) sushi domain/human IgG1 Fc fusion protein, was added. It targets both the innate and adaptive immune system, acting as a growth and activation factor for NK cells as well as effector and memory T cells (18, 19). N-803 alone or in combination with an anti-PD-L1 antibody has been shown to elicit robust anti-tumor immune responses and prolonged survival in tumor-bearing mice (20, 21). N-803 has also been shown to increase PD-L1 expression both *in vitro* (22) and in breast tumor-bearing mice (23); it has been suggested that this may allow lymphocytes to become targets of anti-PD-L1 therapy.

To induce the release of DAMPs, low/moderate dose nab-paclitaxel (Abraxane), paclitaxel bound to albumin that has a better safety profile than paclitaxel and an improved response rate, was used typically several days before the introduction of the PD-L1 t-haNK cells and N-803. Abraxane is used to treat non-small cell lung cancer (NSCLC), breast cancer, and pancreatic cancer and is being studied in other cancers (24–26). Abraxane given together with anti-PD-L1 therapies such as atezolizumab has been reported to prolong progression-free survival among patients with metastatic triple-negative breast cancer (mTNBC) (27) and NSCLC (28). When used at moderate or relatively low doses, Abraxane treatment has the potential to result in the release of tumor cell-associated antigens (TAAs) and DAMPS that may enhance the effects of immunotherapy to elicit a vaccine-like anti-tumor response (29).

Additional experimental chemotherapy was also utilized for the patients described here—aldoxorubicin (ImmunityBio), a doxorubicin prodrug that binds serum albumin post-administration through an acid-sensitive hydrazone linker (30). It leverages the relatively acidic environment of solid tumors that facilitates cleavage of the linker and tumor-targeted delivery of doxorubicin. Aldoxorubicin has shown clinical efficacy and mitigated cardiac toxicity (31–33). In general, for the single-patient Investigational New Drug (spIND) patients here, it was given on the day of PD-L1 t-haNK/N-803 administration.

Some of the patients also received adenovirus vector-based vaccines, including ETBX-051 (Etubics/ImmunityBio), a brachyury vaccine that after subcutaneous administration expresses brachyury that is predicted to elicit a cytotoxic T lymphocyte (CTL)-mediated immune response against tumor cells expressing brachyury and ETBX-061, which, like ETBX-051, is an adenovirus vector-based vaccine that expresses the transmembrane mucin MUC1 (34) after subcutaneous delivery.

Other therapeutics, including cyclophosphamide (an alkylating agent), capecitabine (Xeloda^®^; an anti-metabolite), nivolumab (anti-PD-1), pembrolizumab (anti-PD-1), cisplatin (DNA crosslinker), denosumab (anti-RANKL (35)), and stereotactic body radiation therapy (SBRT; an inducer of DAMP release), were given to individual patients in some instances.

A description of the putative role of each agent in therapy and the manufacturer of each agent are shown in **Supplementary Table S1**.

## Case report 1

**Figure 2:**
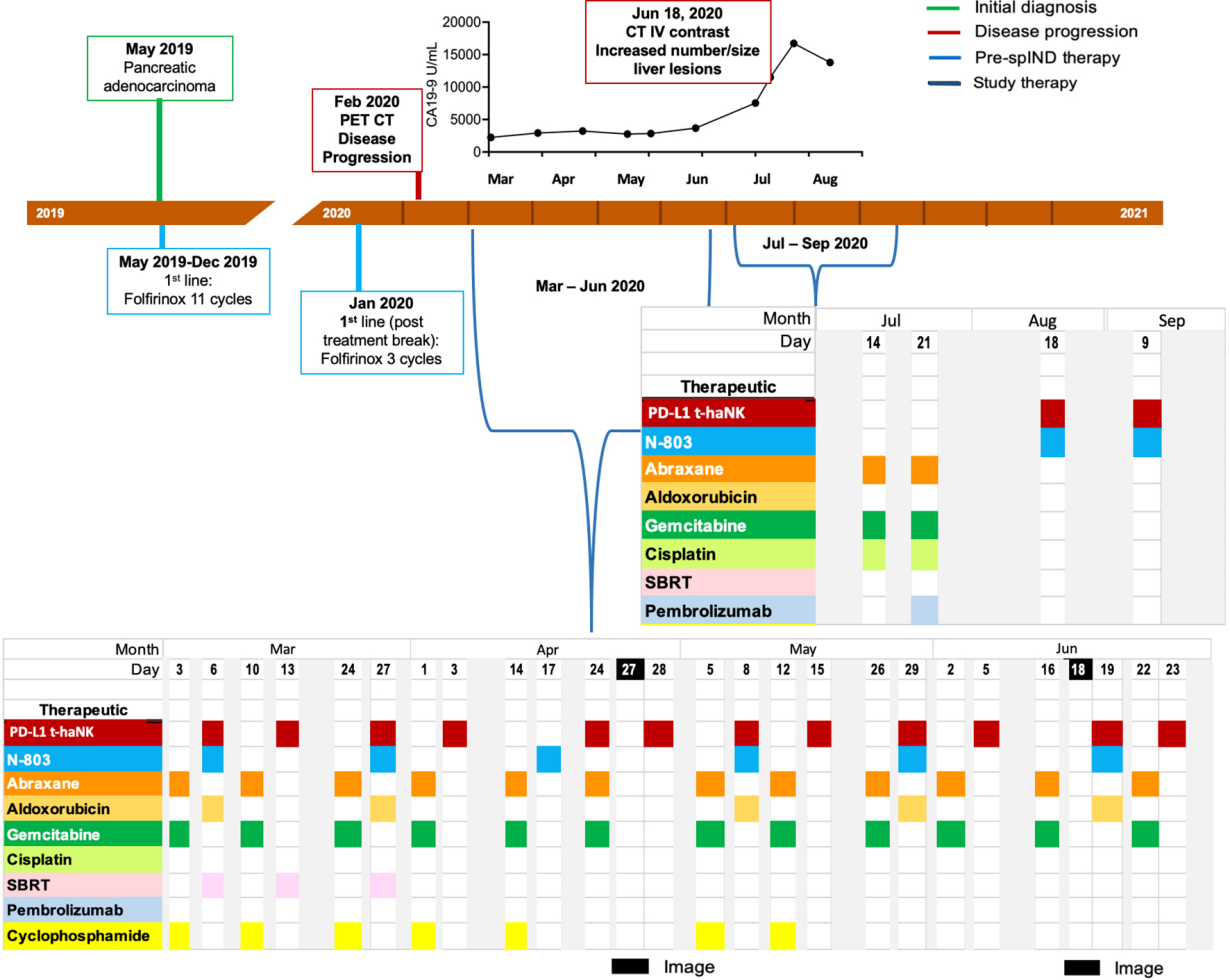
Metastatic pancreatic cancer (mPC) single-patient Investigational New Drug (spIND) patient 1 diagnosis and treatment history. mPC diagnosis (green box) in May 2019 was followed by first-line FOLFIRINOX treatment. Disease progression (red box) detected in February 2020 prompted initiation of a spIND protocol comprising Abraxane (orange), gemcitabine (dark green), cyclophosphamide (light yellow), PD-L1 t-haNK (red), N-803 (aqua), stereotactic body radiation therapy (SBRT; pink), aldoxorubicin (dark yellow), cisplatin (light green), and pembrolizumab (light blue) given on the days indicated. Dates of imaging are shown in black. CA19-9 levels (U/mL) from March to August 2020 are shown above the timeline.

Patient 1 (Pt 1), a 62-year-old man, was diagnosed with mPC in May 2019 (**Supplementary Table S2**) and underwent 11 cycles of first-line FOLFIRINOX therapy (**Figure 2**), a four-drug combination of leucovorin (folinic acid), fluorouracil, irinotecan HCl, and oxaliplatin (36, 37). Initial treatment of this patient was complicated by diarrhea and grade 2 neuropathy. Several months later, the patient received three cycles of FOLFIRINOX as therapy, after which disease progression was observed by PET/CT in February 2020 (**Supplementary Table S2**).

As second-line therapy, a spIND protocol was designed, approved, and consented to by the patient, which commenced in March 2020 and comprised alternating IV gemcitabine (600 mg/m^2^) plus Abraxane (75 mg/m^2^) with IV infusion of 2 × 10^9^ PD-L1 t-haNK cells and 15 μg/kg N-803 by subcutaneous (sc) injection (**Figure 2**). The doses of gemcitabine and Abraxane, respectively, were increased to 750 mg/m^2^ and 100 mg/m^2^ from April 14 to June 22, 2020, and to 1,000 mg/m^2^ and 125 mg/m^2^ on July 14 and 21, 2020. Neutrophil-to-lymphocyte (N/L) ratios varied throughout treatment with a high value recorded on March 13, 2020; the highest absolute neutrophil count (ANC) was observed at baseline on March 3, 2020, and at the last assessment in August 2020 (**Supplementary Table S3**). PET/CT indicated stable disease (SD) in April 2020 (**Table 1**, **Supplementary Table S2**). Along with the first four infusions, the patient received stereotactic body radiation (X-ray) therapy per the schedule shown in **Figure 2**. Aldoxorubicin (80 mg/m^2^) was also given on some days of PD-L1 t-haNK infusion/N-803 delivery and cyclophosphamide (25–50 mg, PO) on some days of Abraxane/gemcitabine delivery. In June, CT indicated progressive disease (PD) (**Supplementary Table S2**). In July 2020, the patient received Abraxane, gemcitabine, and cisplatin (25 mg/m^2^); he reported fevers, rigors, and nausea post-therapy. A single dose of pembrolizumab (200 mg IV) was administered on July 21. In August and September 2020, the patient received PD-L1 t-haNK and N-803 only. CA19-9 levels from March 2020 to August 2020 are shown in **Figure 2** and increased after June 2020, peaking in July 2020. Abraxane and gemcitabine doses were reduced in August due to neutropenia. The patient received spIND treatment for ~6.4 months. The patient’s date of death was March 31, 2021; OS from diagnosis was 22 months, and OS from initiation of spIND therapy was 13 months (**Table 1**).

**Table 1:** Data overview for all patients.

Case study patient #	Line of therapy	Duration of spIND therapy (mo)	Survival from diagnosis	Survival from start of spIND therapy (mo)	Best response	Adverse events during spIND therapy
1	2	6.4	22	13	Stable disease	Post-chemotherapy fever, rigor, nausea, and neutropenia
2	6	3.5	21	4.8	Disease response*	Abdominal distension
3	3	25.4	42	26.9	Biochemical disease response**	Neutropenia, post-chemotherapy fever
4	3	7.4	13	9	Disease response*	Neutropenia, anemia
5	4	23.2	33	23.2	Complete response	Fatigue, bilateral leg edema, neutropenia, anemia

spIND, single-patient Investigational New Drug (regimen); mo, months.

*Disease response, reduction in target lesion size, not necessarily meeting Response Evaluation Criteria in Solid Tumors (RECIST) criteria for a defined partial response.

**Disease response suggested by decreased CA19-9 levels.

## Case report 2

**Figure 3:**
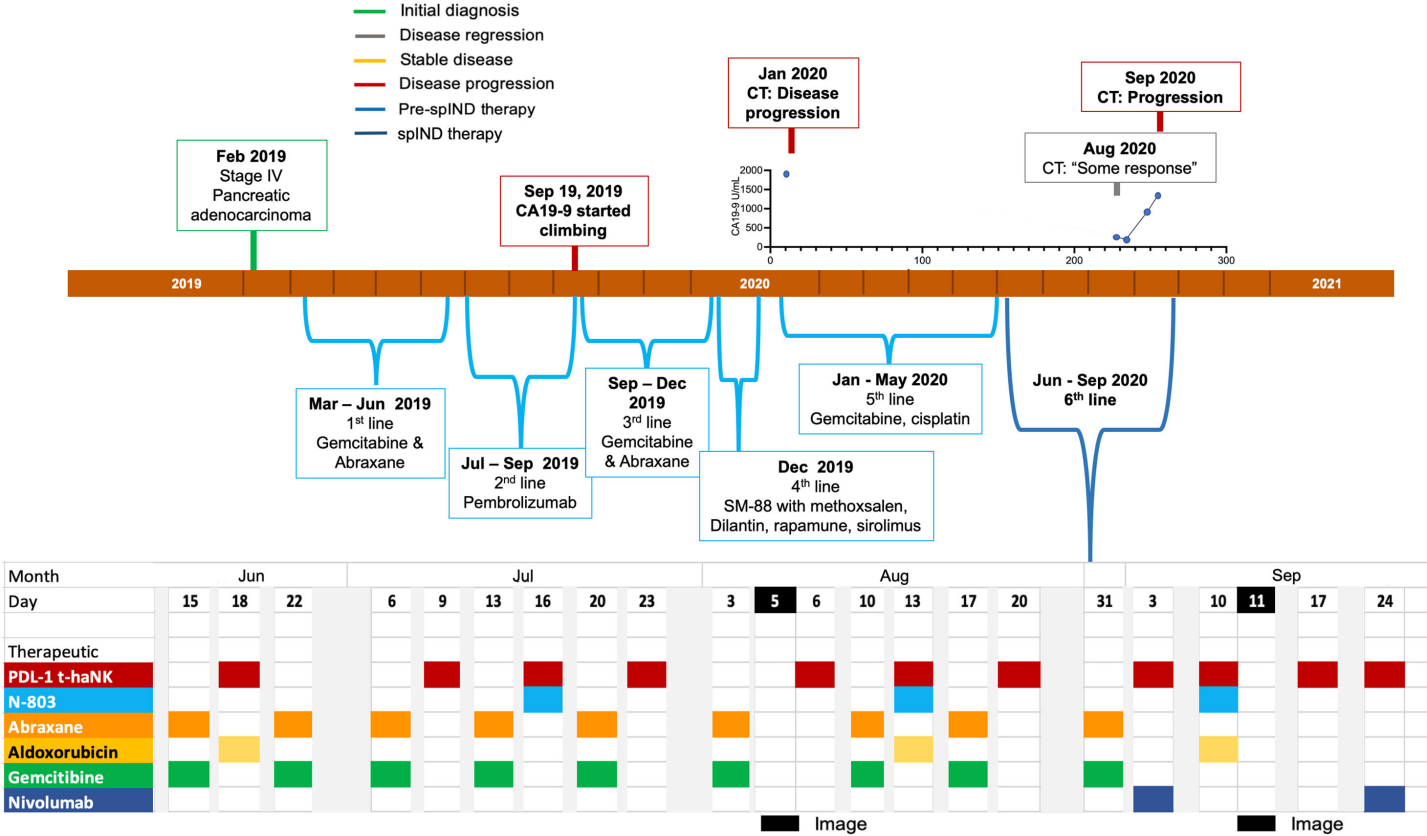
Metastatic pancreatic cancer (mPC) single-patient Investigational New Drug (spIND) patient 2 diagnosis and treatment history. mPC diagnosis (green box) in February 2019 was followed by first-, second-, third-, fourth- (experimental SM-88 therapy), and fifth-line therapies up to June 2020, at which time the spIND protocol commenced comprising Abraxane (orange), gemcitabine (green), PD-L1 t-haNK (red), N-803 (blue), aldoxorubicin (yellow), and nivolumab (dark blue) given on the days indicated. There was evidence of disease progression (red boxes) and some response (gray box) on the dates shown. Dates of imaging are shown in black. CA19-9 levels (U/mL) in January 2020 and from August to September 2020 are shown above the timeline.

Patient 2 (Pt 2), a 78-year-old man, was diagnosed with stage IV pancreatic adenocarcinoma in February 2019 (**Supplementary Table S2**) and received gemcitabine and Abraxane as first-line treatment (**Figure 3**). Due to the toxicity of first-line therapy, the patient was switched to pembrolizumab as second-line therapy in July 2019. In response to increasing CA19-9 levels in September 2019, gemcitabine and Abraxane therapy was reinstated. In December 2019, the patient was given an experimental regimen of SM-88 with methoxsalen, Dilantin, Rapamune, and sirolimus as fourth-line therapy. CA19-9 levels were relatively elevated in January 2020, and a CT scan revealed progressive disease (**Supplementary Table S2**); the patient began treatment with a combination of gemcitabine and cisplatin.

In June 2020 as sixth-line therapy, a spIND protocol comprising PD-L1 t-haNK (IV infusion 2 × 10^9^ cells/dose), N-803 (15 μg/kg sc), and aldoxorubicin (150 mg/m^2^ IV) alternating with IV gemcitabine (300 mg/m^2^) and Abraxane (50 mg/m^2^) was initiated with the patient’s consent, with therapeutics delivered as shown in **Figure 3**. The lowest ANC was observed at baseline in June 2020 and the highest at the last assessment in September 2020; N/L values were lower throughout treatment relative to baseline (**Supplementary Table S4**). Nivolumab (480 mg) was added and gemcitabine/Abraxane was withheld starting in September 2020. During the course of spIND therapy, the patient continued to report abdominal distension. The patient received spIND treatment for 3.5 months. In August 2020, a CT scan showed some disease response (**Table 1**, **Supplementary Table S2**) at the same time CA19-9 levels were low; disease progression and CA19-9 level increases were seen in September 2020 (**Figure 3**, **Supplementary Table S2**). The patient’s date of death was November 8, 2020. The patient received spIND therapy for 3.5 months, survival from diagnosis was 21 months, and survival from initiation of spIND therapy was 4.8 months (**Table 1**).

## Case report 3

Patient 3 (Pt 3), a 78-year-old man, was diagnosed with grade III pancreatic adenocarcinoma in May 2018 (**Supplementary Table S2**) at which time he underwent a distal pancreatectomy and splenectomy. First-line therapy from July to October 2018 was FOLFIRINOX with Neulasta (**Supplementary Figure S1**). A PET/CT in May 2019 revealed metastases (**Supplementary Table S2**); CA19-9 levels also rose from ~40 U/mL in February 2019 to 4,484 U/mL in August 2019. As second-line therapy, Abraxane and gemcitabine were given between July and September 2019. spIND therapy was initiated in November 2019 with the patient’s consent and comprised PD-L1 t-haNK (2 × 10^9^ cells/infusion IV), aldoxorubicin (80 mg/m^2^), cyclophosphamide (50–75 mg PO), N-803 (15 μg/kg sc), Abraxane (75 mg/m^2^), and gemcitabine (600 mg/m^2^) with a single instance of denosumab (60 mg) administration up to the end of 2019 (**Supplementary Figure S1**).

In December 2019, the chemotherapeutic agents were held due to neutropenia.

The patient’s therapy continued from January to June 2020, including the therapeutics described above, as well as a single dose of capecitabine (500 mg PO) (**Supplementary Figure S2**). In February 2020, immunotherapy was held due to fever post-chemotherapy, and in April 2020, treatment was delayed for ANC. The dose of aldoxorubicin was decreased to 60 mg/m^2^ in March. Abraxane and gemcitabine were held for several months starting in May. CA19-9 levels over this treatment period remained under 1,000 U/mL (**Supplementary Figure S2**). N/L and ANC values varied throughout therapy, as shown in **Supplementary Table S5**.

**Figure 4:**
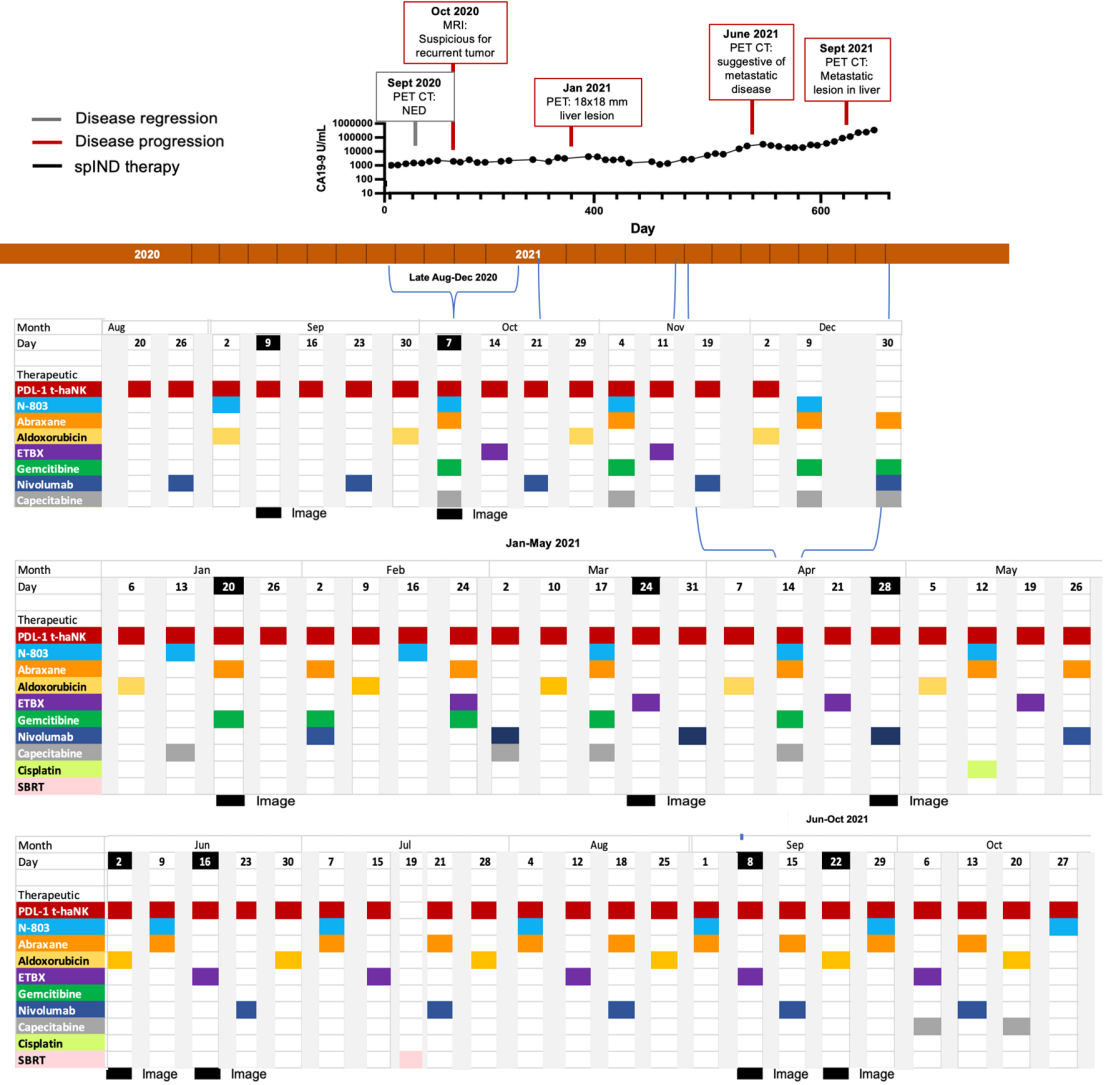
Metastatic pancreatic cancer (mPC) single-patient Investigational New Drug (spIND) patient 3 treatment history from late August 2020 until October 2021. The spIND protocol comprised PD-L1 t-haNK (red), N-803 (blue), Abraxane (orange), aldoxorubicin (yellow), ETBX vaccines 051 and 061 (purple), gemcitabine (green), nivolumab (dark blue), and capecitabine (gray), given on the dates shown; the patient also received a single dose of cisplatin (light green) and a single instance of stereotactic body radiation therapy (SBRT; pink). Disease regression is shown in gray boxes, disease progression in red boxes, and dates of imaging in black. CA19-9 levels (U/mL) from September 2020 to October 2021 are shown above the timeline.

Beginning in late August 2020, nivolumab (480 mg IV) was added to the therapeutic arsenal for patient 3, and Abraxane/gemcitabine was re-introduced in October 2020 (**Figure 4**). Also in October 2020, the patient received vaccination with ETBX-051/ETBX-61 [both 1 × 10^11^ viral particles (VP) sc], and dosing with capecitabine (500 mg PO) became more frequent. This regimen continued until October 2021 and included a single administration of cisplatin (25 mg/m^2^) in May 2021 and a single instance of SBRT in July 2021. As shown in **Figure 4**, CA19-9 levels surpassed the 1,000 U/mL level in September and remained steady until May/June, at which time they began to rise. This increase was associated with evidence of progressive disease (**Supplementary Table S2**). The patient received 25.4 months of spIND treatment (up to October 2021), after which he received palliative care and died in January 2022. He survived 42 months from the time of diagnosis and ~27 months from initiation of spIND therapy (**Table 1**).

## Case report 4

Patient 4 (Pt 4), a 59-year-old woman, was diagnosed with pancreatic adenocarcinoma in November 2019 (**Supplementary Table S2**) and received first-line FOLFIRINOX plus CPI from November 2019 to February 2020 treatment, during which time (January 2020) pancreatic disease appeared stable (**Supplementary Table S2**) but suspicious liver lesions were detected. In March 2020, second-line treatment with 5-FU, gemcitabine, and Abraxane was given (**Supplementary Figure S3**). In April 2020, with the patient’s consent, third-line spIND treatment was initiated with PD-L1 t-haNK (4 × 10^9^ cells IV), N-803 (15 μg/kg sc), gemcitabine (600 mg/m^2^), and Abraxane (100 mg/m^2^ starting; then decreased to 75 mg/m^2^) with two rounds of SBRT, intermittent cyclophosphamide (50 mg PO), and aldoxorubicin administration (150 mg/m^2^); and addition of nivolumab (480 mg) in September 2020 (**Figure 4**). The highest N/L ratio and ANC were recorded near the end of spIND therapy (**Supplementary Table S6**). Some response was observed in June 2020, but new liver metastases were observed in October 2020 (**Supplementary Table S2**). The patient received single doses of ETBX-05 and ETBX-061 vaccines (1 × 10^11^ VP sc) in November. Therapy lasted for 7.4 months and continued until December 2020. Over the last 6 months of therapy, the patient showed weight loss. The patient’s date of death was January 21, 2021; OS from diagnosis was 13 months, and OS from initiation of spIND therapy was 9 months (**Table 1**).

## Case report 5

Patient 5 (Pt 5), a 61-year-old woman, was diagnosed with pancreatic cancer in March 2019 (**Supplementary Table S2**), and from April to December 2019, she underwent first-line therapy with FOLFIRINOX, followed by second-line radiation therapy with Xeloda and third-line gemcitabine and Abraxane therapy, as shown in **Supplementary Figure S4**. Disease progression was detected by a laparoscopic look in December 2019 and a slight decrease in lesion size by PET/CT in January 2020 (**Supplementary Table S2**), at which time the highest ANC value was observed (**Supplementary Table S7**). As fourth-line therapy, spIND therapy was initiated in January 2020 with the patient’s consent and comprised Day 1 gemcitabine (750 mg/m^2^ starting, decreased to 600 or 300 mg/m^2^ on some occasions, IV) and Abraxane (100 mg/m^2^ starting and then decreased to 75 mg/m^2^, IV) followed by Day 3 PD-L1 t-haNK cells (2 × 10^9^ cells IV), N-803 (15 μg/kg sc), and aldoxorubicin (80 mg/m^2^ starting and then decreased to 40–60 mg/m^2^) on a 1-week cycle until September 2020. CA19-9 levels in July and August 2020 were 47 and 117 U/mL, respectively; PET/CT and CT with IV contrast suggested that the disease was stable during this period (**Supplementary Table S2**). Neutropenia was noted early in the therapeutic regimen (February 2020) and anemia in April 2020.

From October 2020 to December 2021 (**Supplementary Figure S5**), gemcitabine and Abraxane were not given, and dosing with other therapeutics was less frequent. ETBX-051/ETBX-061 vaccines (1 × 10^11^ VP sc) were added during this time period. In March 2021, a PET/CT revealed no evidence of active malignancy (**Supplementary Table S2**). In May 2021, regular ~2-week cycles of PD-L1 t-haNK with either the vaccines or aldoxorubicin were established, with the addition of N-803 in August through November. spIND therapy lasted 23.2 months. CA19-9 levels did not vary greatly from January to October 2021 and were low, ranging from 36 to 117 U/mL. Some fatigue was reported by the patient in October 2021 and bilateral leg edema. Disease progression—a liver lesion—was assessed by PET/CT in August 2021 (**Supplementary Table S2**). The patient was still alive as of the writing of this manuscript, with an OS from diagnosis of 33 months and OS from initiation of spIND therapy of 23.2 months (**Table 1**).

## Discussion

We chose the unusual approach of presenting several case studies in a single report to enable better representation and understanding of the orchestrated, multi-modal therapy strategy employed for these advanced PC patients. Considered together, these case studies reveal the potential for this strategy to extend the survival of this patient group.

The OS of the five patients treated by orchestrated multi-modal nab-paclitaxel/gemcitabine, N-803, PD-L1 t-haNK cell, and aldoxorubicin-based therapy, whether from the commencement of spIND therapy (range 4.8 to 26.9 months) or diagnosis (range 13–42 months), is notable and highly clinically relevant (**Table 1**). Perhaps more remarkable is that therapy was the second line for only one of these patients, the third line for two, and the fourth or sixth line for one each.

Because the line of therapy was greater than second in four of five of the case studies here, it is difficult to compare OS to published reports, but if one considered studies of second-line therapy such as that of Tsang et al., a median OS of ~8 months was observed with gemcitabine/nab-paclitaxel (38) in patients who were, as compared to those who did not receive second-line therapy, were younger, with lower Eastern Cooperative Oncology Group (ECOG), and with higher CA19-9 (at presentation). In another study (39), the median OS for FOLFOX as second-line therapy was 2.6–6.7 months, and that for 5-FU/leucovorin plus nanoliposomal irinotecan was ~6 months. In the sole second-line patient presented here, OS was 13 months from the start of spIND therapy and 22 months from diagnosis. The shortest OS here of 4.8 months from the start of spIND therapy was for the sixth-line patient.

The compassionate use of spIND therapy was well-tolerated, with patients reporting few adverse events. Those that were noted—fatigue, fevers, rigors, nausea, and abdominal distension—or based on laboratory assessments (neutropenia and anemia) were not unexpected and likely associated with chemotherapy.

While the individual spIND protocols were not designed so that regular assessments of tumor size or metastases would be evaluated by, for example, Response Evaluation Criteria in Solid Tumors (RECIST) criteria, imaging scans suggested that at some point during spIND therapy, stable disease was achieved in patient 1; disease response in patients 2, 3, and 4; and a complete response in patient 5 (**Table 1**). ANC levels and N/L ratios did not show a notable pattern among patients; although higher values are associated with decreased overall survival in cancer (40) and with the presence of metastases in pancreatic cancer (41), the relationship between these values and response to therapy is not fully understood.

The findings presented here provide support for larger trials of multi-modal therapy that includes PD-L1 t-haNK cells, N-803, and other therapeutics that play a role in inducing immunogenic cell death. Such clinical trials may be limited to patients who have failed first-line or greater therapy, but there may also be merit in determining the benefit in patients as first-line therapy. In such future studies, it would be of interest to collect pre-study tumor tissue biopsies for gene expression and other analyses to allow identification of the TME as well as tumor cell molecular and genetic factors that are associated with response, duration, or failure of this treatment regimen.

## Data Availability

The original contributions presented in the study are included in the article/**Supplementary Material**. Further inquiries can be directed to the corresponding author.
