# Supplementary material for: Case report: PD-L1-targeted high-affinity natural killer cells and IL-15 superagonist N-803-based therapy extend overall survival of advanced metastatic pancreatic cancer patients
Source: Front Oncol. 2025 Jan 29;15:1472714. doi: 10.3389/fonc.2025.1472714 (PMC11813753; doi:10.3389/fonc.2025.1472714)
Supplement: Supplementary file 1 [file DataSheet1.pdf]

Supplementary Material for:

**Case Report: PD-L1 Targeted High-Affinity Natural Killer Cell and IL-15 Superagonist N-803-Based Therapy Extend Overall Survival of Advanced Metastatic Pancreatic Cancer Patients**

Tara Seery, Lennie Sender, Omid Jafari, Frank Jones, Patricia Spilman, Sandeep B. Reddy, Patrick Soon-Shiong

**Supplementary Figures**......2
**Figure S1**. Patient 3 May 2018-Dec 2019......2
**Figure S2**. Patient 3 Jan-Aug 2020......3
**Figure S3.** Patient 4 Diagnosis & Treatment History......4
**Figure S4**. Patient 5 Mar 2019-Sep 2020......5
**Figure S5**. Patient 5 Oct 2020-Dec 2021......6
**Supplementary Tables** ......7
**Table S1.** Agents and Role in Therapy......7
**Table S2.** Tumor Assessment (PET, CT, MRI)......8
**Table S3**. Laboratory Investigations Patient 1......9
**Table S4**. Laboratory Investigations Patient 2......9
**Table S5**. Laboratory Investigations Patient 3......10
**Table S6**. Laboratory Investigations Patient 4......11
**Table S7**. Laboratory Investigations Patient 5......12

## Supplementary Figures

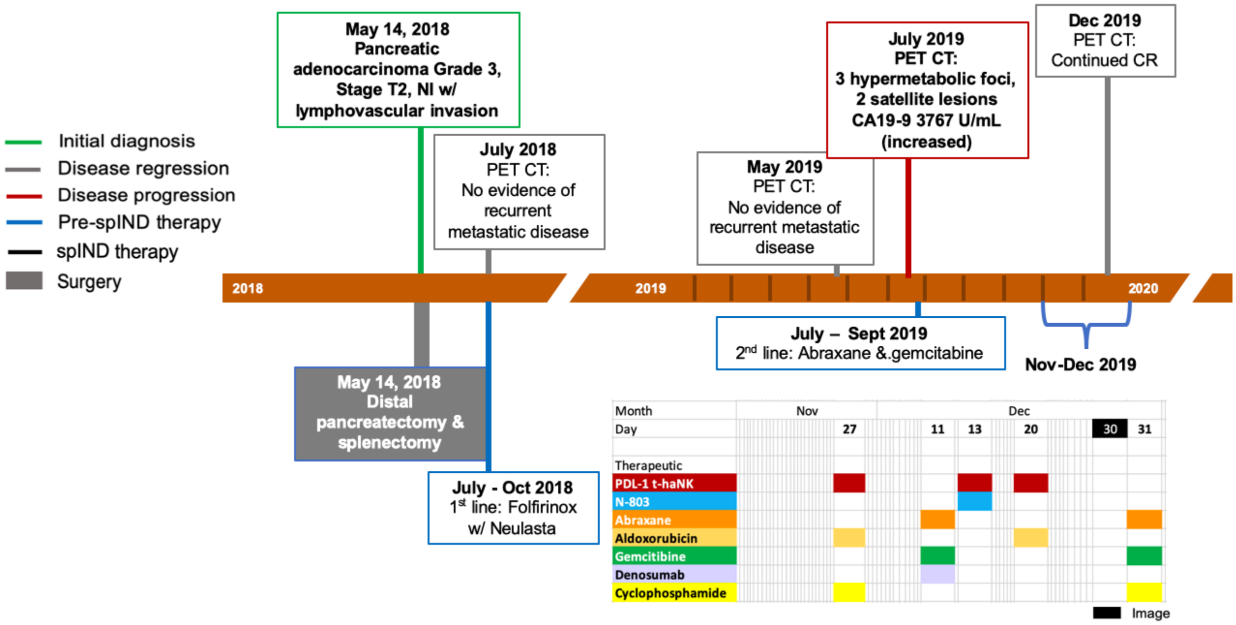


**Figure S1**. *mPC spIND patient 3 diagnosis and treatment until the end of 2019*. At the time of pancreatectomy and splenectomy in May 2018, the patient was diagnosed with pancreatic adenocarcinoma. He received FOLFIRINOX plus Neulasta and later Abraxane plus gemcitabine before and spIND therapy was initiated in November 2019. The spIND protocol comprised PD-L1 t-haNK (red), N-803 (blue), aldoxorubicin (yellow), Abraxane (orange), gemcitabine (green), cyclophosphamide (light yellow), given on the dates shown; the patient also received two doses of denosumab (light purple). Disease regression is shown in gray boxes, and progression in red boxes on the dates shown. Dates of imaging are shown in black.

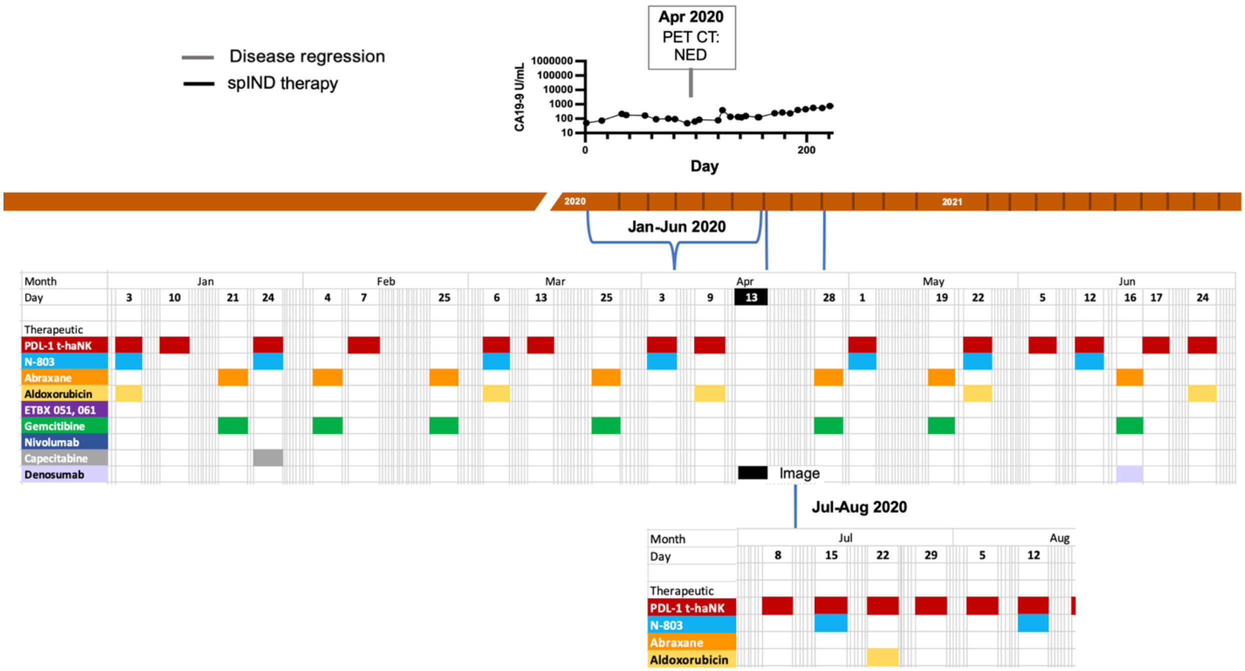


**Figure S2.** *mPC spIND patient 3 treatment from January to August 2020*. The spIND protocol comprised PD-L1 t-haNK (red), N-803 (blue), Abraxane (orange), aldoxorubicin (yellow), gemcitabine (green), capecitabine (gray), given on the dates shown; the patient also received a single dose of denosumab (light purple). Disease regression is shown in gray boxes and dates of imaging are shown in black. CA19-9 levels (U/mL) from January to August 2020 are shown above the timeline.

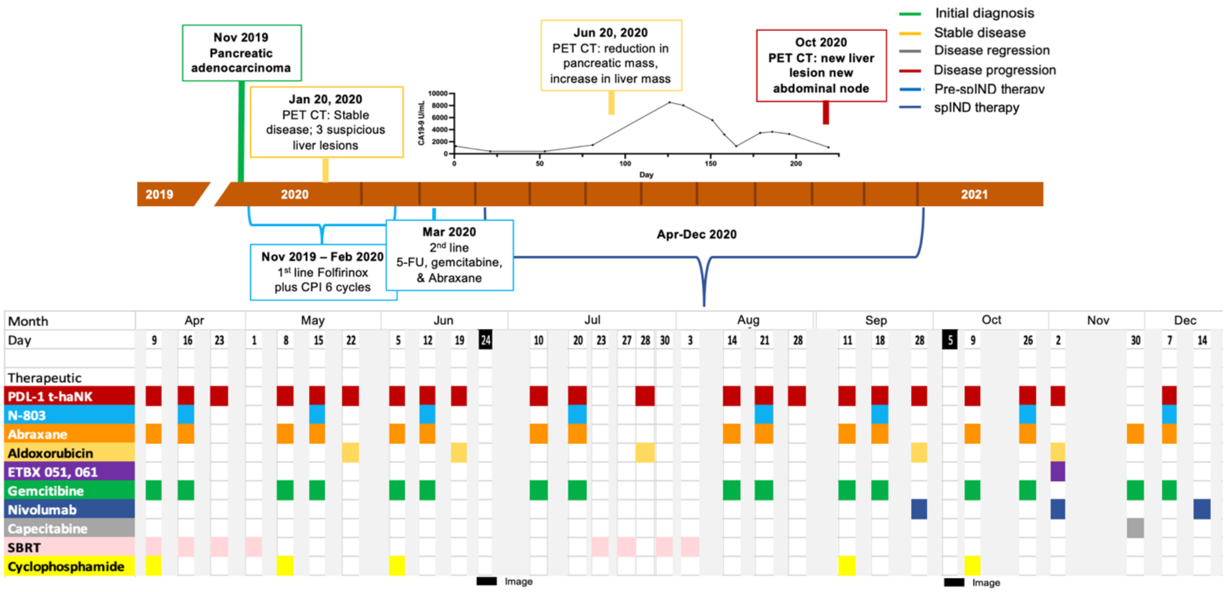


**Figure S3**. *mPC spIND patient 4 diagnosis and treatment history*. After initial diagnosis of pancreatic adenocarcinoma in November 2019, the patient received 1st and 2nd line therapy as indicated. In April 2020, spIND therapy comprising PD-L1 t-haNK (red), N-803 (blue), aldoxorubicin (yellow), Abraxane (orange), gemcitabine (green), capecitabine (single administration; gray), cyclophosphamide (light yellow), ETBX -051/-061 vaccines (single administration; purple), nivolumab (dark blue), and stereotactic body radiation therapy (SBRT, pink). PET CT indicating stable disease is shown in yellow boxes and disease progression in a red box. Dates of imaging are shown in black. CA19-9 levels (U/mL) from March to October 2020 are shown above the timeline.

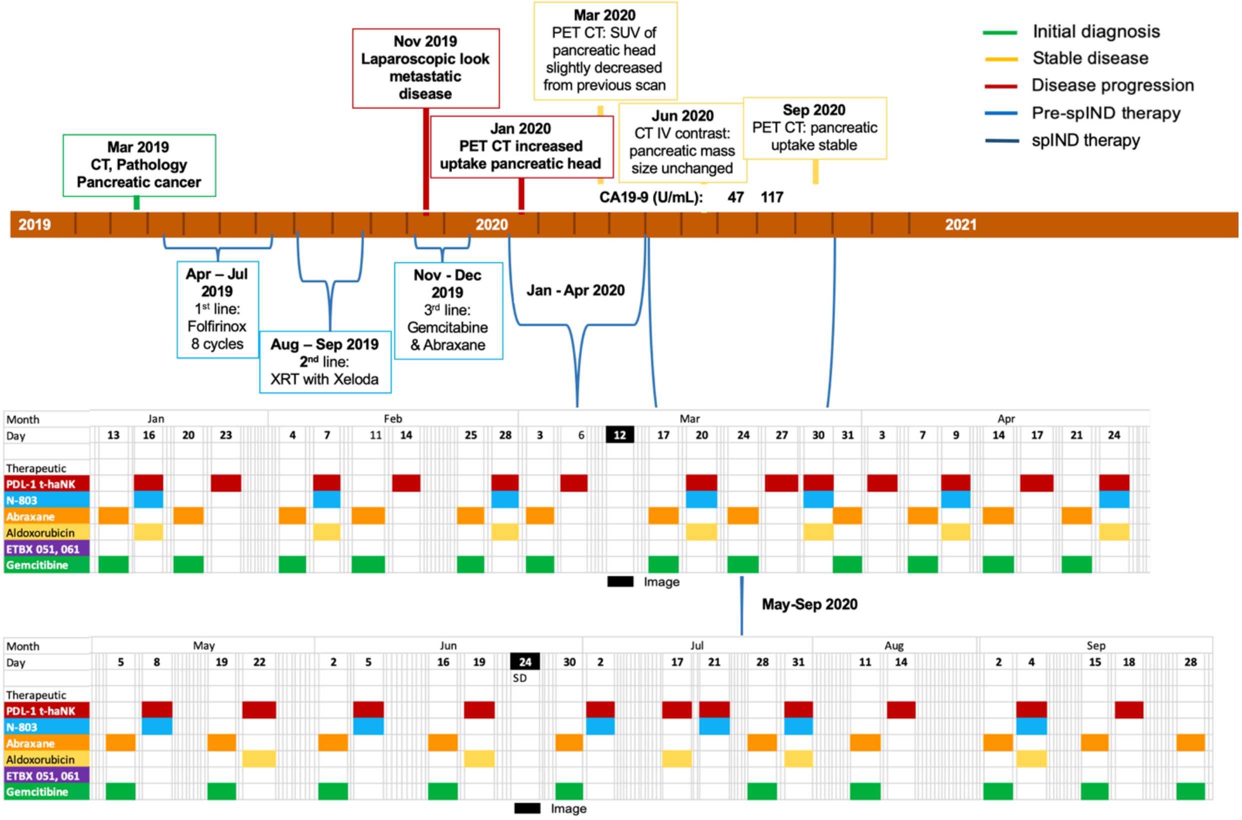


**Figure S4.** *mPC spIND patient 5 diagnosis and treatment from March to September 2020.* After initial diagnosis of pancreatic adenocarcinoma in March 2019, the patient received 1st and 2nd line therapy as indicated. In January 2020, spIND therapy comprising PD-L1 t-haNK (red), N-803 (blue), Abraxane (orange), aldoxorubicin (yellow), and gemcitabine (green) was initiated as shown. Laparoscopy or PET CT indicating stable disease (yellow boxes) or disease progression (red boxes) are shown. Dates of imaging are shown in black. CA19-9 levels (U/mL) values for July and August 2020 are shown above the timeline.

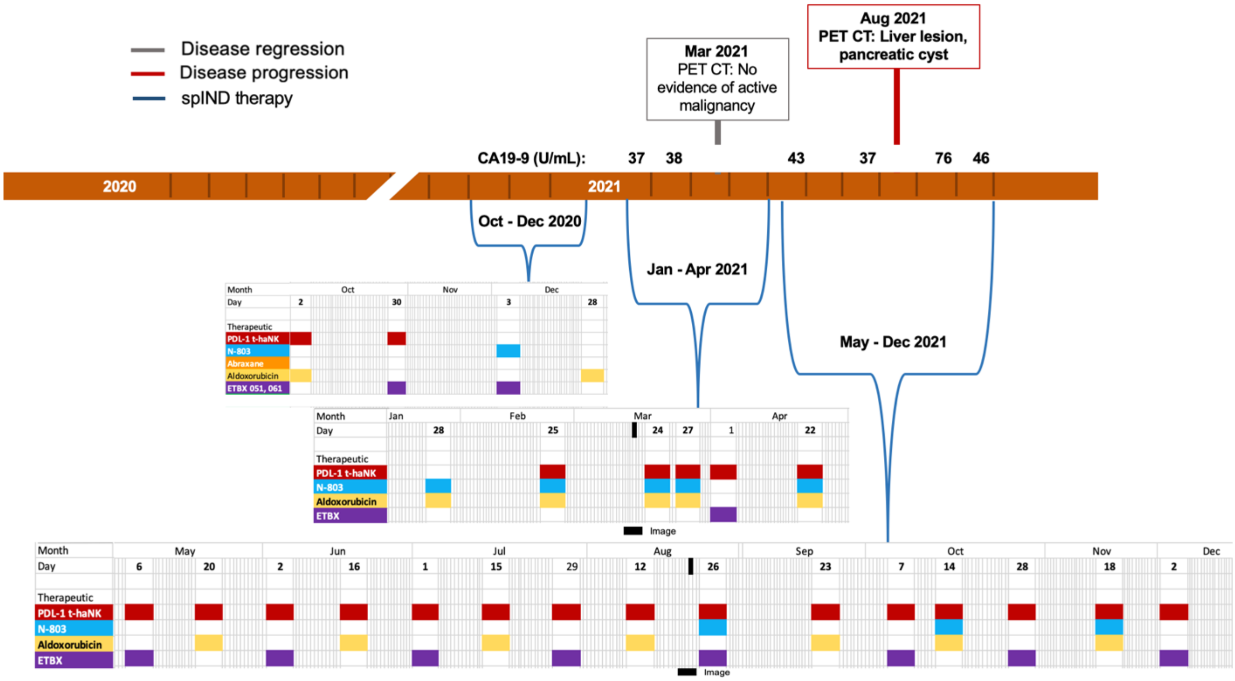


**Figure S5.** *mPC spIND patient 5 treatment from October 2020 to December 2021*. From October 2020 on, the patient received only PDL1 t-haNK (red), N-803 (blue), aldoxorubicin (yellow), and ETBX -051/-061 vaccines (purple). PET CT indicating disease regression (gray box) or disease progression (red boxes) are shown. Dates of imaging are shown in black. CA19-9 levels (U/mL) values are listed above the timeline.

## Supplementary Tables

| Table S1. Agents and Role in Therapy | | | | | | | | |
|---|---|---|---|---|---|---|---|---|
| | | Role | | | | | | |
| **Agent** | **Description** | **Tumor cell cytotoxicity** | **Release of TAAs** | **Coordinate ICD signals** | **Mitagate TME immuno-suppression** | **Enhance immune responses** | **Condition dendritic & T cells** | **Maintain immune responses** |
| **PDL-1 t-haNK*** | Off-the- Shelf Programmed Death Receptor Ligand 1 Targeted High Affinity Natural Killer Cells | | | | | | | |
| **N-803*** | Nogapendekin alfa inbakicept (Anktiva®) interleukin-15 (IL-15) superagonist | | | | | | | |
| **Abraxane** | Nab-Paclitaxel, albumin-bound taxane | | | | | | | |
| **Aldoxorubicin*** | Doxorubicin conjugate with acid-sensitive linker | | | | | | | |
| **Capecitabine** | Fluorouracil pro-drug inhibitor of DNA synthesis | | | | | | | |
| **Cyclophosphamide** | DNA crosslinker inhibits protein synthesis | | | | | | | |
| **Gemcitabine** | Deoxycytidine analog inhibitor of DNA synthesis | | | | | | | |
| **Cisplatin** | DNA crosslinker inhibits protein synthesis | | | | | | | |
| **SBRT** | Stereotactic Body Radiation Therapy | | | | | | | |
| **Pembrolizumab** | mAb against programmed death receptor 1 (PD-1) | | | | | | | |
| **Nivolumab** | mAb against programmed death receptor 1 (PD-1) | | | | | | | |
| **ETBX 051, 061**** | Adenovirus-vectored tumor antigen-targeted vaccines; 051- brachyury, 061 - MUC1 | | | | | | | |
| *ImmunityBio, Inc. investigational agent; **Etubics (wholly owned by ImmunityBio, Inc.) investigational agents; TAAs - Tumor-associated antigens; ICD. - Immunogenic cell death; TME - Tumor microenvironment | | | | | | | | |

| Table S2. Tumor Responses | | | | | |
|---|---|---|---|---|---|
| **Date** | **Preceding Rx** | **Finding** | **PET** | **Diagnostic CT** | **MRI** |
| **Patient 1** | | | | | |
| May-19 | Diagnosis | Pancreatic cancer (method not indicated) | | | |
| Feb-20 | FOLFIRINOX | Disease advancement | | X | |
| Apr-20 | spIND | No change (stable) | X | X | |
| Jun-20 | spIND | Disease advancement | | X | |
| **Patient 2** | | | | | |
| Feb-19 | Diagnosis | Pancreatic cancer | | X | |
| Jan-20 | SM-88 | Disease advancement | | X | |
| Aug-20 | spIND | Decrease in lesion size | | X | |
| Sep-20 | spIND | New foci | | X | |
| **Patient 3** | | | | | |
| May-18 | Diagnosis | Pancreatic cancer | | X | X |
| May-19 | FOLFIRINOX | Disease advancement | X | X | |
| Sep-20 | spIND | Small liver foci | | X | |
| Oct-20 | spIND | Liver lesion | | X | |
| Jan-21 | spIND | Liver lesion | X | | |
| Mar-21 | spIND | Disease advancement (liver) | | | X |
| Apr-21 | spIND | hypoenhancing region | | | X |
| Jun-21 | spIND | possible recurrence | | | X |
| Jun-21 | spIND | New metastatic disease | X | X | |
| Sep-21 | spiND | New liver met; resolved previous | X | X | |
| **Patient 4** | | | | | |
| Nov-19 | Diagnosis | Pancreatic cancer | | X | X |
| Jan-20 | FOLFIRINOX | No change (stable) | | X | |
| Jun-20 | spIND | Decrease in lesion size | X | X | |
| Oct-20 | spIND | New liver met | X | X | |
| **Patient 5** | | | | | |
| Mar-19 | Diagnosis | Pancreatic cancer | | X | |
| Nov-19 | FOLFIRINOX | Liver metastases (laparoscopy look) | | | |
| Jan-20 | spIND | Slight decrease lesion size | X | X | |
| Mar-20 | spIND | Stable; some decrease lesion size | X | X | |
| Jun-20 | spIND | Stable | | X | |
| Sep-20 | spIND | Stable | X | X | |
| Mar-21 | spIND | No evidence of disease; complete response | X | X | |
| Aug-21 | spIND | 9 mm liver lesion | X | X | |

| Table S3. Laboratory Investigations Patient 1 | | | | | | | |
|---|---|---|---|---|---|---|---|
| **Date** | **WBC** | **Neut #** | **Lymph #** | **N/L** | **Hgb** | **Plt** | **ANC** |
| **3/3/20** | **5.10** | **3.29** | **1.05** | **3.13** | **14.0** | **210** | **3.29** |
| 3/10/20 | 2.30 | 1.58 | 0.36 | 4.39 | 13.4 | 99 | 1.48 |
| 3/13/20 | 6.90 | 6.52 | 0.18 | **36.22** | 11.4 | 101 | |
| 3/24/20 | 4.80 | 2.43 | 1.20 | 2.03 | 13.1 | 425 | 2.43 |
| 4/1/20 | 2.80 | 1.44 | 0.46 | 3.13 | 13.2 | 169 | 1.44 |
| 4/14/20 | 4.20 | 2.01 | 0.93 | 2.16 | 12.5 | 207 | 2.01 |
| 4/24/20 | 4.60 | 2.36 | 0.83 | 2.84 | 11.8 | 149 | 2.36 |
| 5/5/20 | 5.30 | 2.97 | 1.00 | 2.97 | 11.2 | 220 | 2.97 |
| 5/11/20 | | | | | 10.4 | 131 | 1.40 |
| 5/15/20 | 2.91 | 2.40 | 0.27 | **8.89** | 9.4 | 123 | 2.40 |
| 5/26/20 | 4.30 | 2.63 | 0.56 | 4.70 | 10.5 | 329 | 2.63 |
| 6/2/20 | 2.52 | 1.47 | 0.29 | 5.07 | 10.9 | 201 | 1.47 |
| 6/16/20 | 5.43 | 2.70 | 0.66 | 4.09 | 10.1 | 298 | 2.70 |
| 6/22/20 | 2.30 | 1.12 | 0.18 | **6.22** | 10.1 | 221 | 1.12 |
| 7/14/20 | 4.47 | 1.93 | 0.97 | 1.99 | 11.5 | 293 | 1.93 |
| 7/21/20 | 2.79 | 1.43 | 0.57 | 2.51 | 11.9 | 150 | 1.43 |
| 8/18/20 | 5.12 | 3.33 | 0.70 | 4.76 | 10.8 | 53 | **3.23** |

WBC - white blood cells; Neut - neutrophils; Lymph - lymphocytes; N/L - neutrophils/lymphocytes; Hgb – hemoglobin; Plt - platelets; ANC - Absolute neutrophil count. Baseline values in bold. Relatively high neutrophil-to-lymphocyte ratios (N/L) and absolute neutrophil counts (ANC) in red.

| Table S4. Laboratory Investigations Patient 2 | | | | | | | |
|---|---|---|---|---|---|---|---|
| **Date** | **WBC** | **Neut #** | **Lymph #** | **N/L** | **Hgb** | **Plt** | **ANC** |
| **6/18/20** | **4.49** | **2.71** | **1.24** | **2.19** | **10.7** | **237** | **1.25** |
| 6/25/20 | 3.80 | 2.20 | 1.10 | 2.00 | 10.6 | 117 | 2.20 |
| 7/9/20 | 5.81 | 2.92 | 1.80 | 1.62 | 11.3 | 313 | 2.92 |
| 7/16/20 | 5.31 | 2.64 | 2.08 | 1.27 | 12 | 276 | 2.64 |
| 8/13/20 | 5.90 | 2.96 | 2.00 | 1.48 | 11.4 | 164 | 2.96 |
| 9/10/20 | 6.17 | 3.33 | 1.84 | 1.81 | 11.9 | 162 | **3.33** |

WBC - white blood cells; Neut - neutrophils; Lymph - lymphocytes; N/L - neutrophils/lymphocytes; Hgb - hemoglobin: Plt - platelets; ANC - Absolute neutrophil count. Baseline values in bold. Relatively high absolute neutrophil count (ANC) in red.

**Table S5. Laboratory Investigations Patient 3**

| Date | WBC | Neut # | Lymph # | N/L | Hgb | Plt | ANC | | Date | WBC | Neut # | Lymph # | N/L | Hgb | Plt | ANC |
|---|---|---|---|---|---|---|---|---|---|---|---|---|---|---|---|---|
| **9/10/19** | **12.60** | **10.15** | **1.71** | **5.94** | **11.0** | **372** | | | 2/2/21 | 3.57 | 0.74 | 0.97 | 0.76 | 10.1 | 176 | 0.74 |
| 1/24/20 | 4.90 | 2.00 | 1.40 | 1.43 | 11.3 | 342 | 2.00 | | 2/9/21 | 2.58 | 1.34 | 0.70 | 1.91 | 9.5 | 150 | 1.34 |
| 2/7/20 | 4.80 | 1.20 | 1.80 | 0.67 | 10.3 | 198 | 1.53 | | 2/16/21 | 2.63 | 0.70 | 0.84 | 0.83 | 9.7 | 174 | 0.70 |
| 3/6/20 | 4.00 | 1.80 | 0.80 | 2.25 | 9.4 | 149 | 1.80 | | 2/24/21 | 5.16 | 2.40 | 0.93 | 2.58 | 10.1 | 252 | 2.40 |
| 4/9/20 | 3.19 | 1.54 | 0.32 | **4.81** | 9.2 | 177 | 1.54 | | 3/2/21 | 2.79 | 1.61 | 0.66 | 2.44 | 9.6 | 96 | 1.61 |
| 5/1/20 | 3.40 | 1.10 | 0.80 | 1.38 | 10.1 | 346 | 1.10 | | 3/10/21 | 3.16 | 0.66 | 0.87 | 0.76 | 10.3 | 191 | 0.66 |
| 6/12/20 | 4.24 | 2.06 | 0.84 | 2.45 | 10.5 | 262 | 2.06 | | 3/17/21 | 5.57 | 2.79 | 1.19 | 2.34 | 10.9 | 290 | 2.79 |
| 6/24/20 | 3.06 | 1.49 | 0.77 | 1.94 | 10.1 | 97 | 1.49 | | 3/24/21 | 2.9 | 1.82 | 0.61 | 2.98 | 9.5 | 86 | 1.82 |
| 7/22/20 | 4.13 | 3.06 | 0.43 | **7.12** | 9.5 | 101 | **3.06** | | 3/31/21 | 3.05 | 0.41 | 1.09 | 0.38 | 9.8 | 213 | 0.41 |
| 7/29/20 | 2.86 | 1.00 | 0.74 | 1.35 | 10.3 | 183 | 1.00 | | 4/7/21 | 5.12 | 2.54 | 1.21 | 2.10 | 10.1 | 323 | 2.54 |
| 8/20/20 | 4.16 | 2.74 | 0.64 | **4.28** | 9.7 | 93 | 2.74 | | 4/14/21 | 5.47 | 2.78 | 1.16 | 2.40 | 10.2 | 252 | 2.78 |
| 9/2/20 | 2.69 | 1.77 | 0.49 | 3.61 | 9.9 | 238 | 1.77 | | 4/21/21 | 3.62 | 2.36 | 0.69 | 3.42 | 9.1 | 80 | 2.36 |
| 9/30/20 | 3.27 | 1.07 | 0.73 | 1.47 | 10.0 | 149 | 1.07 | | 4/28/21 | 3.15 | 0.62 | 1.04 | 0.60 | 9.6 | 228 | 0.62 |
| 10/7/20 | 3.24 | 1.73 | 0.69 | 2.51 | 10.5 | 239 | 1.73 | | 5/5/21 | 4.26 | 1.82 | 1.13 | 1.61 | 10.2 | 312 | 1.82 |
| 10/20/20 | 2 | 0.2 | 0.8 | 0.25 | 9.5 | 130 | 0.20 | | 5/12/21 | 5.94 | 3.28 | 1.26 | 2.60 | 10.8 | 222 | **3.28** |
| 10/29/20 | 3.6 | 1.2 | 1.1 | 1.09 | 10.3 | 310 | 1.20 | | 5/19/21 | 3.74 | 1.74 | 1.06 | 1.64 | 9.7 | 144 | 1.74 |
| 11/4/20 | 4.3 | 2 | 1 | 2.00 | 10.2 | 214 | 2.00 | | 5/26/21 | 3.65 | 1.49 | 1.11 | 1.34 | 10.1 | 206 | 1.49 |
| 11/10/20 | 1.7 | 0.7 | 0.6 | 1.17 | 8.7 | 83 | 0.70 | | 6/2/21 | 2.95 | 1.6 | 1.03 | 1.55 | 9.9 | 155 | 1.60 |
| 11/19/20 | 4.1 | 0.5 | 1.5 | 0.33 | 9.7 | 186 | 0.50 | | 6/9/21 | 3.35 | 1.17 | 1.33 | 0.88 | 9.9 | 154 | 1.17 |
| 12/2/20 | 5.6 | 1.8 | 1.9 | 0.95 | 9.6 | 252 | 1.80 | | 6/16/21 | 1.82 | 0.82 | 0.78 | 1.05 | 9.1 | 144 | 0.82 |
| 12/9/20 | 5.2 | 2.2 | 1.7 | 1.29 | 9.9 | 202 | 2.20 | | 6/23/21 | 3.08 | 0.42 | 1.24 | 0.34 | 8.8 | 215 | 0.42 |
| 12/30/20 | 4.6 | 1.4 | 1.6 | 0.88 | 9.5 | 310 | 0.40 | | 6/30/21 | 7.03 | 4.55 | 1.25 | 3.64 | 8.7 | 214 | **4.55** |
| 1/6/21 | 2.3 | 0.9 | 1.1 | 0.82 | 9.5 | 114 | 0.90 | | 7/7/21 | 4.7 | 2.97 | 0.97 | 3.06 | 8.0 | 172 | 2.97 |
| 1/13/21 | 3.2 | 0.8 | 1.2 | 0.67 | 9.4 | 157 | 0.80 | | 9/8/21 | 3.36 | 1.74 | 1.01 | 1.72 | 8.3 | 174 | 1.74 |
| 1/20/21 | 4.6 | 1.8 | 1.2 | 1.50 | 9.5 | 269 | 1.80 | | 9/15/21 | 3.34 | 1.12 | 1.11 | 1.01 | 8.5 | 192 | 1.12 |
| 1/26/21 | 3.4 | 2.4 | 0.6 | 4.00 | 9.5 | 155 | 2.40 | | 9/22/21 | 4.59 | 2.9 | 1.07 | 2.71 | 8.4 | 174 | 2.90 |

WBC - white blood cells; Neut - neutrophils; Lymph - lymphocytes; N/L - neutrophils/lymphocytes; Hgb - hemoglobin; Plt - platelets; ANC - Absolute neutrophil count. Baseline in bold. Relatively high neutrophil-to-lymphocyte ratios (N/L) and absolute neutrophil counts (ANC) in red.

| Table S6. Laboratory Investigations Patient 4 | | | | | | | |
|---|---|---|---|---|---|---|---|
| **Date** | **WBC** | **Neut #** | **Lymph #** | **N/L** | **Hgb** | **Plt** | **ANC** |
| **4/9/20** | **2.39** | **1.28** | **0.64** | **2.00** | **9.4** | **120** | **1.28** |
| 4/16/20 | 1.77 | 1.00 | 0.59 | 1.69 | 8.9 | 90 | 1.00 |
| 4/23/20 | 1.30 | 0.44 | 0.46 | 0.96 | 8.1 | 101 | 0.44 |
| 4/30/20 | 4.00 | | | | 9.2 | 618 | |
| 5/8/20 | 4.49 | 3.03 | 0.57 | 5.32 | 9.0 | 362 | 3.03 |
| 5/15/20 | 3.66 | 2.73 | 0.29 | 9.41 | 8.0 | 138 | 2.73 |
| 5/22/20 | 3.24 | 1.93 | 0.36 | 5.36 | 8.4 | 124 | 1.93 |
| 6/5/20 | 2.25 | 1.24 | 0.37 | 3.35 | 9.0 | 259 | 1.24 |
| 6/12/20 | 2.40 | 1.81 | 0.17 | **10.65** | 7.8 | 164 | 1.81 |
| 6/19/20 | 2.06 | 1.43 | 0.15 | 9.53 | 8.2 | 115 | 1.43 |
| 7/10/20 | 4.89 | 2.58 | 1.46 | 1.77 | 8.6 | 300 | 2.58 |
| 7/20/20 | 3.09 | 1.82 | 0.62 | 2.94 | 8.6 | 159 | 1.82 |
| 7/28/20 | 3.33 | 1.5 | 0.82 | 1.83 | 8.1 | 136 | 1.50 |
| 8/14/20 | 2.19 | 0.97 | 0.51 | 1.90 | 8.6 | 212 | 0.97 |
| 8/21/20 | 2.65 | 1.95 | 0.28 | 6.96 | 8.1 | 141 | 1.95 |
| 8/28/20 | 2.82 | 1.63 | 0.34 | 4.79 | 7.2 | 65 | 1.63 |
| 9/11/20 | 4.1 | 1.12 | 0.81 | 1.38 | 8.2 | 247 | 1.12 |
| 9/18/20 | 2.83 | 1.65 | 0.53 | 3.11 | 8.0 | 127 | 1.65 |
| 10/9/20 | 3.48 | 2.11 | 0.42 | 5.02 | 10.0 | 318 | 2.11 |
| 10/26/20 | 6.26 | 4.53 | 0.34 | **13.32** | 10.7 | 129 | **4.53** |
| 10/30/20 | 2.89 | 2.34 | 0.27 | 8.67 | 10.9 | 210 | 2.34 |
| 11/25/20 | 6.5 | 5.21 | 0.53 | 9.83 | 8.3 | 138 | **5.21** |
| 12/2/20 | 14.72 | 14.22 | 0.18 | **79.00** | 7.4 | 143 | |
| 12/14/20 | 1.39 | 1.1 | 0.23 | 4.78 | 5.3 | 30 | 2.88 |

WBC - White blood cells; Neut - Neutrophils; Lymph - Lymphocytes; Hgb - Hemoglobin; Plt - Platelets; ANC - Absolute neutrophil count. Baseline in bold. Relatively high neutrophil-to-lymphocytes ratios (N/L) absolute neutrophil count (ANC) in red.

| Table S7. Laboratory Investigations Patient 5 | | | | | | | |
|---|---|---|---|---|---|---|---|
| Date | WBC | Neut # | Lymph # | N/L | Hgb | Plt | ANC |
| **1/12/20** | **9.10** | | | | **9.7** | **147** | **7.92** |
| 1/19/20 | 3.10 | | | | 8.8 | 245 | |
| 2/3/20 | | | | | 9.8 | 225 | 2.90 |
| 2/10/20 | 1.1 | 0.65 | 0.13 | 5.00 | 9.0 | 117 | |
| 2/14/20 | 5.9 | 2.86 | 0.75 | 3.81 | 8.6 | 90 | 2.86 |
| 2/24/20 | 5.5 | 2.57 | 0.72 | 3.57 | 9.9 | 266 | 2.57 |
| 3/2/20 | 1.41 | 1.02 | 0.11 | 9.27 | 8.8 | 97 | 1.02 |
| 3/6/20 | 2.4 | 1.73 | 0.14 | **12.36** | 7.3 | 57 | 1.73 |
| 3/15/20 | 3.8 | 2.05 | 0.44 | 4.66 | 7.9 | 138 | 2.05 |
| 3/20/20 | 1.9 | 1.27 | 0.12 | 10.58 | 8.7 | 213 | 1.27 |
| 4/6/20 | 2.20 | 1.64 | | | 7.7 | 187 | 1.64 |
| 4/9/20 | 2.30 | 1.87 | 0.15 | **12.47** | 7.8 | 111 | 1.87 |
| 4/20/20 | 3.60 | 1.95 | | | 6.3 | 187 | 1.95 |
| 5/4/20 | 4.10 | 2.2 | 0.4 | 5.50 | 9.8 | 137 | 2.20 |
| 6/29/20 | 3.60 | 1.75 | 0.32 | 5.47 | 9.3 | 123 | 1.75 |
| 7/27/20 | 5.70 | 2.98 | 0.64 | 4.66 | 9.0 | 257 | 2.98 |
| 8/14/20 | 4.60 | 3.22 | 0.56 | 5.75 | 7.8 | 148 | 3.20 |
| 8/31/20 | 5.30 | 2.69 | 1.44 | 1.87 | 9.7 | 238 | 2.96 |
| 9/14/20 | 4.90 | 3.35 | 0.55 | 6.09 | 8.6 | 126 | 3.35 |
| 9/30/20 | 4.70 | | | | 7.9 | 153 | 3.25 |
| 10/27/20 | 6.7 | 4.83 | 0.58 | 8.33 | 7.7 | 235 | 4.83 |
| 12/28/20 | 7.5 | 5.65 | 0.85 | 6.65 | 8.1 | 165 | 5.65 |
| 1/28/21 | 7.6 | 6.55 | 0.42 | **15.60** | 8.3 | 114 | **6.55** |
| 2/22/21 | 4.2 | 3.06 | 0.59 | 5.19 | 5.7 | 195 | 3.52 |
| 3/24/21 | 4.4 | 2.81 | 0.7 | 4.01 | 8.9 | 204 | 2.81 |
| 4/20/21 | 6 | 3.91 | 0.94 | 4.16 | 9.3 | 237 | 3.91 |
| 5/4/21 | 6.9 | 4.33 | 1.14 | 3.80 | 6.7 | 234 | 4.33 |
| 5/18/21 | 5.4 | 3.24 | 0.93 | 3.48 | 7.9 | 241 | 3.24 |
| 6/14/21 | 5.8 | 3.83 | 0.89 | 4.30 | 5.9 | 228 | 3.83 |
| 6/28/21 | 6.9 | 4.99 | | | 8.4 | 244 | 4.99 |
| 7/15/21 | 5.7 | 4.03 | 0.75 | 5.37 | 7.3 | 185 | |
| 7/29/21 | 5.1 | 3.68 | 0.62 | 5.94 | 7.1 | 187 | |
| 8/11/21 | 4.8 | 3.36 | 0.64 | 5.25 | 7.8 | 180 | |
| 9/21/21 | 5.4 | 3.2 | 1.03 | 3.11 | 8.8 | 237 | |
| WBC - White blood cells; Hbg - hemoglobin; Plt - Platelets; ANC - Absolute neutrophil count. Baseline in bold. Relatively high neutrophil-to-lymphocyte ratios (N/L) and absolute neutrophil count (ANC) in red. | | | | | | | |
